# The glycine brace: a component of Rab, Rho, and Ran GTPases associated with hinge regions of guanine- and phosphate-binding loops

**DOI:** 10.1186/1472-6807-9-11

**Published:** 2009-03-05

**Authors:** Andrew F Neuwald

**Affiliations:** 1 Institute for Genome Sciences and Department of Biochemistry & Molecular Biology, University of Maryland School of Medicine, 801 West Baltimore St., BioPark II, Room 617, Baltimore, MD 21201, USA

## Abstract

**Background:**

Ras-like GTPases function as on-off switches in intracellular signalling pathways and include the Rab, Rho/Rac, Ran, Ras, Arf, Sar and Gα families. How these families have evolutionarily diverged from each other at the sequence level provides clues to underlying mechanisms associated with their functional specialization.

**Results:**

Bayesian analysis of divergent patterns within a multiple alignment of Ras-like GTPase sequences identifies a structural component, termed here the glycine brace, as the feature that most distinguishes Rab, Rho/Rac, Ran and (to some degree) Ras family GTPases from other Ras-like GTPases. The glycine brace consists of four residues: An aromatic residue that forms a stabilizing CH-π interaction with a conserved glycine at the start of the guanine-binding loop; a second aromatic residue, which is nearly always a tryptophan, that likewise forms stabilizing CH-π and NH-π interactions with a glycine at the start of the phosphate-binding P-loop; and two other residues (typically an aspartate and a serine or threonine) that, together with a conserved buried water molecule, form a network of interactions connecting the two aromatic residues.

**Conclusion:**

It is proposed that the two glycine residues function as hinges and that the glycine brace influences guanine nucleotide binding and release by interacting with these hinges.

## Background

Rab [1], Rho/Rac [2] and Ran [3,4] GTPases regulate diverse cellular processes including vesicle trafficking, cytoskeletal dynamics, cell polarity, membrane fusion, chromosome segregation, and nuclear transport. These proteins are a subgroup of the extended Ras-like superfamily of GTPases [5] (termed here the Ras-like GTPases), which function as signaling pathway on-off switches and which also include Arf, Arf-like (Arl), and Sar GTPases and α subunits of heterotrimeric G proteins. Given an appropriate upstream signal, Ras-like GTPases are turned 'on' by binding to GTP, resulting in their association with various 'effectors' that propagate the incoming signal to downstream components. Guanine nucleotide exchange factors (GEFs) facilitate this process by mediating the exchange of GTP for GDP. Ras-like GTPases are turned off upon hydrolysis of GTP to GDP, which results in termination of the signal and a shutting off of the pathway. GTPase activating proteins (GAPs) facilitate this process by stimulating the inherent GTPase activity of Ras-like GTPases.

**Figure 1:**
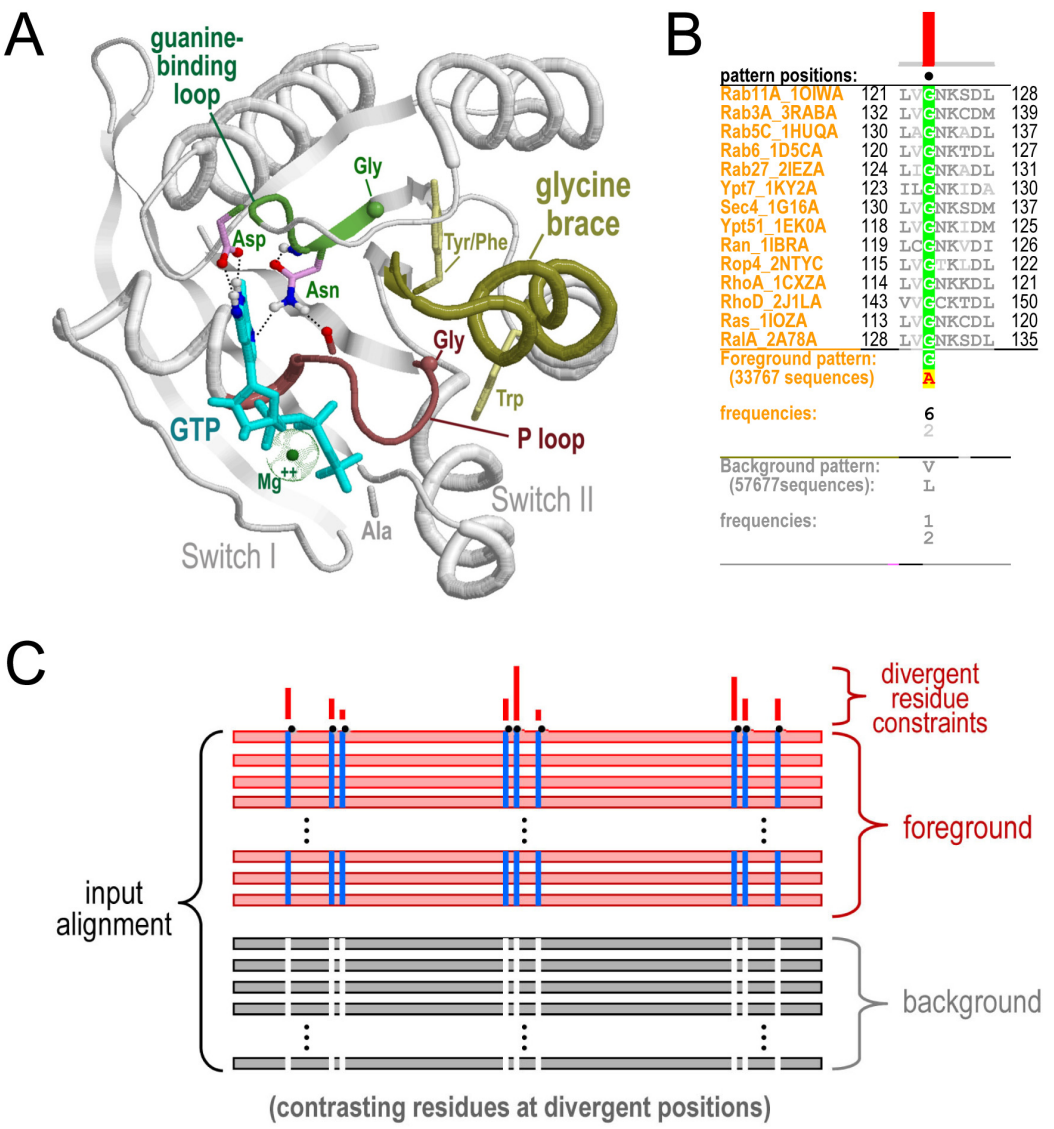
**Attributes and analysis of Rab-related and Ras-like GTPases**. **(A) **Relevant structural features of Rab-related GTPases. The structure shown is that of Sec4 bound to a GTP analogue (pdb_id: 1g17)[34]. The two glycine residues proposed to function as hinges are indicated (with their α-carbon atoms displayed as spheres). Also indicated are: a conserved alanine residue, repositioning of which is proposed to facilitate nucleotide exchange by occluding the Mg^++ ^binding site [13]; the conserved asparagine and aspartate residues of the guanine-binding loop; and the conserved aromatic residues of the glycine brace. **(B) **A contrast alignment (defined in (C)) highlighting a conserved residue position near the guanine-binding loop that (along with other conserved residues outside of the region shown) distinguishes all Ras-like GTPases (the 'foreground') from other P-loop GTPases (the 'background'). Relevant foreground and background consensus residues at this position, along with corresponding (weighted) residue frequencies, are shown directly below the alignment of representative GTPase sequences (protein names and pdb identifiers are indicated in the leftmost column). Residue frequencies are given in integer tenths where, for example, a '6' indicates that the corresponding residue occurs in 60%–70% of the sequences. **(C) **Schematic representation of a contrast alignment [12,31]. A contrast alignment is the output returned by the CHAIN program's [12] BPPS procedure [11], which optimally partitions an input alignment into 'foreground' and 'background' sub-alignments such that the foreground sequences (red horizontal bars) strikingly conserve a pattern that is non-conserved in (or that contrasts with) the background sequences (gray horizontal bars) at pattern positions (blue vertical bars). The red vertical bars over pattern positions quantify the selective pressure (as previously defined [11]) that is imposed on the foreground relative to the background.

Ras-like GTPases are a subgroup of the phosphate-binding loop (P-loop) GTPases, which bind GTP via amino acid residues corresponding to several conserved motifs [6]. Two of these motifs are relevant to the analysis here: the Walker A (G-x_4_-G-K-[ST]) motif [7], which corresponds to the P-loop [8], and a [NT]-K-x-D motif, which occurs within the guanine-binding loop. The residues of the Walker A motif bind to the phosphate groups of GDP or GTP, whereas the residues of the guanine-binding motif bind to the guanine base and link it to the P-loop [9,10]. Here I identify a structural component, termed the glycine brace, that is specifically conserved in Rab, Ran and Rho/Rac GTPases and that spans these two guanine nucleotide binding regions (Figure 1A).

## Results

### Analysis of Ras-like GTPases

**Figure 2:**
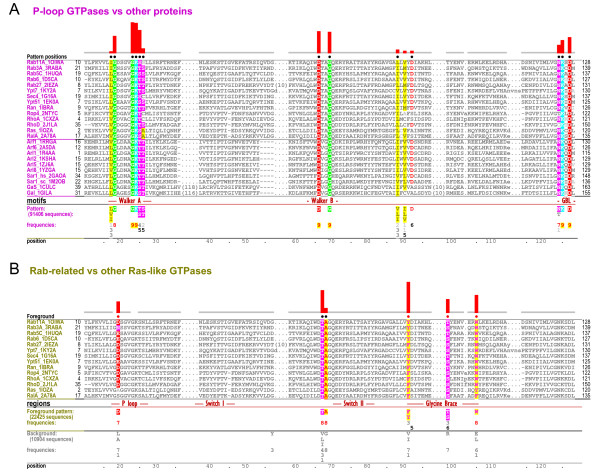
**Contrast alignments showing the most distinctive sequence features of P-loop GTPases and of Rab-related GTPases**. See legends to Figures 1B,C for descriptions. **(A) **Distinctive sequence features of P-loop GTPases. The acronym 'GBL' stands for guanine-binding loop. **(B) **Distinctive sequence features of Rab-related GTPases. The foreground (Rab-related GTPases) includes Rab, Ran, Rho and some (but not all) Ras family GTPases; the background consists of other Ras-like GTPases.

Using a 'Bayesian partitioning with pattern selection' (BPPS) procedure [11], a multiple alignment of 91,406 P-loop GTPase sequences (the most conserved features of which are shown in Figure 2A) was optimally partitioned into two major subgroups – Ras-like GTPases versus other P loop GTPases – based on amino acid differences at evolutionarily-divergent residue positions. This identified about a dozen residue positions that are conserved in Ras-like GTPases but not in other P-loop GTPases and that thus presumably reflect characteristic features shared by these on-off switches. Figure 1B highlights one of these conserved residues that is relevant to the analysis here – namely a glycine or alanine immediately preceding the guanine-binding loop; the other residues will be described elsewhere (Neuwald, unpublished).

The alignment in Figure 1B, which is termed a 'contrast alignment', corresponds to the output produced by the BPPS procedure (although with only a short region relevant to this analysis shown here). As described in Figure 1C, a contrast alignment consists of two contrasting sub-alignments, one of which contains strikingly conserved patterns that are non-conserved in the other sub-alignment. Thus the highlighted glycine or alanine in Figure 1B is specifically conserved within Ras-like GTPases, implying that it performs a function specific to these proteins. The analysis here provides some clues regarding this function – at least for those Ras-like GTPases that typically conserve a glycine at this position.

### Rab-related GTPases

**Figure 3:**
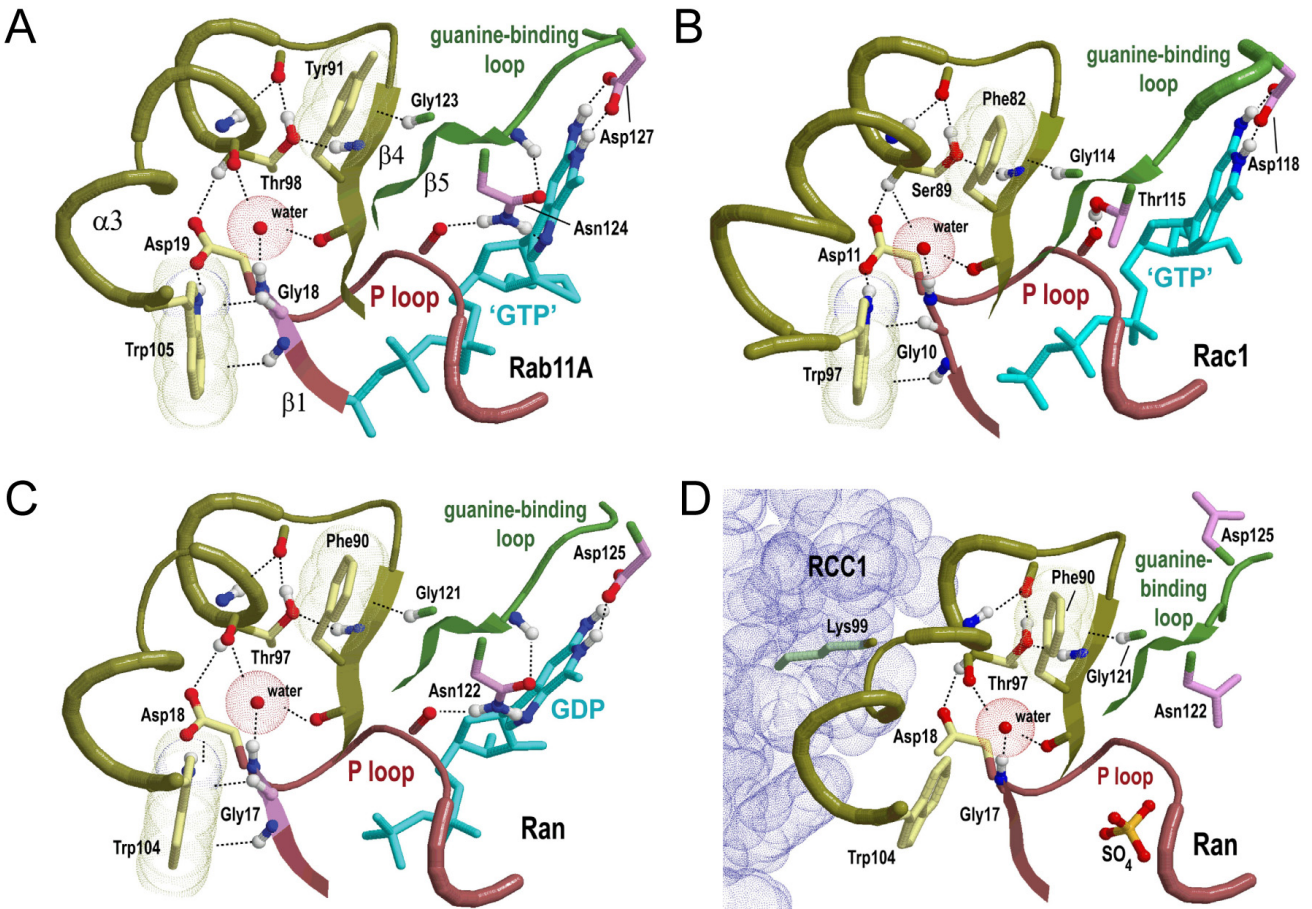
**The glycine brace within crystal structures of representative Rab-related GTPases**. Color scheme: backbone of the P-loop region, dark red; backbone of the glycine brace region, dark yellow; backbone of the guanine-binding loop region, green; side chains of residues characteristic of P-loop GTPases, magenta; side chains of residues characteristic of the Rab-related GTPases, yellow. **(A) **The Rab-family GTPase Rab11A bound to a GTP analog (GTPγS) (pdb_id: 1oiw)[35]. **(B) **The Rho/Rac family GTPase Rac1 bound to a GTP analog (pdb_id: 1i4t) [36]. **(C) **Ran GTPase bound to GDP (pdb_id: 1byu) [37]. **(D) **Ran GTPase bound to its exchange factor RCC1 (pdb_id: 1i2m) [20].

Application of the BPPS procedure [11,12] to a sub-alignment consisting of Ras-like GTPases (an abridged version of which is shown in Figure 1B) resulted in a natural partitioning into two major Ras-like GTPase subgroups: Rab, Ran, Rac/Rho and certain Ras GTPases (the Rab-related subgroup) versus other Ras-like GTPases. The corresponding contrast alignment is shown in Figure 2B. The associated pattern corresponds to six conserved residue positions that can be split into two groups: (i) the two adjacent residue positions (a threonine and an alanine) located between the Walker B aspartate and the glycine at the start of the switch II region; and (ii) four residue positions that structurally correspond to the glycine brace (Figure 3). The alanine residue within the first group is proposed to play a role in nucleotide exchange by pushing out the Mg++ ion that coordinates with the guanine nucleotide phosphates [13].

The four pattern residues corresponding to the glycine brace include: (i) a conserved acidic or amidic residue ([DENQ]) immediately following the conserved glycine at the start of the P-loop (Asp19-Rab11A in Figures 2B and 3A); (ii) a tyrosine or phenylalanine ([YF]) (Tyr91-Rab11) within the β4-strand, which forms a β-sheet with the β-strand directly preceding the guanine-binding loop; (iii) a serine or threonine ([ST]) (Thr98-Rab11) within the a3-helix; and (iv) a tryptophan ([W])(or, rarely, a tyrosine or phenylalanine) (Trp105-Rab11A) also within the α3-helix. Rab, Ran and Rho GTPases generally conserve all of these patterns, but members of the Ras family typically lack matches to the canonical pattern at one or two positions and thus appear to have undergone additional evolutionary divergence. Despite these divergent features, however, by and large the Ras-family is still classified by the BPPS procedure into the Rab-related subgroup.

### The glycine brace

The analysis in Figure 2B indicates that the most distinctive feature of Rab-related GTPases is the glycine brace (Figure 3), which is structurally characterized by nearly a dozen conserved atomic interactions. One of these is a CH-π interaction [14] between the aromatic residue corresponding to the [FY] pattern (Phe91-Rab11A in Figure 3A) and the conserved glycine (Gly123-Rab11A in Figures 1B and 3A) within the adjacent parallel β-strand that immediately precedes the guanine-binding loop. The presence of glycine intrinsically destabilizes β sheets, but this sort of aromatic-glycine interaction has been proposed to counteract this effect [15]. Thus, within Rab-related GTPases, this CH-π interaction could stabilize the region directly preceding the guanine-binding loop, which conserves residues (Figure 2A) that bind both to the guanine base and to the P-loop (Figure 3).

Likewise, the tryptophan residue of the glycine brace (Trp105-Rab11A) often forms both a NH-π [16] and a CH-π interaction with main-chain atoms of a glycine that is located at the start of the P-loop (Gly18-Rab11A in Figures 2A and 3A) and that is highly conserved in P-loop GTPases (about 90% of the sequences in Figure 2A) and very highly conserved within glycine-brace GTPases (over 99% of the foreground sequences in Figure 2B).

Two other glycine brace residues, an acidic or amidic residue (Asp19-Rab11A in Figures 2B and 3A) and a serine or threonine residue (T98-Rab11A), can participate in a network of hydrogen bonds linking the two aromatic residues associated with the P-loop and with the guanine-binding-loop. A buried water molecule, which is conserved across nearly all Rab-related GTPase crystal structures, also participates in this interaction network (Figure 3). In contrast, other Ras-like GTPases (i.e., members of the Arf, Arl, Sar and Gα families) are characterized by a strikingly different network of interactions (unpublished observations).

### Non-glycine residues preceding the guanine-binding loop

Ninety-five percent of the sequences classified in this analysis as glycine-brace-containing GTPases (Figure 2B) harbor a glycine residue immediately preceding the guanine-binding loop. Many of the remaining glycine brace GTPases (3.7%) harbor an alanine instead of a glycine at this position, whereas the rest (1.4%) harbor some other residue. These non-glycine variants still conserve the four-residue pattern associated with the glycine brace, and for variants of known structure the glycine brace phenylalanine or tyrosine still forms a CH-π interaction with the backbone α-carbon hydrogen atom just as for typical glycine brace GTPases. These include three alanine variants: human RhoB (pdb_id: 2fv8)(Structural Genomics Consortium)(SGC), mouse M-ras; (pdb_id: 1x1r) [17], and Rab5a from *Plasmodium falciparum *(pdb_id: 3clv)(SGC). This also includes one glutamine variant: mouse Rab23 (pdb_ids: 1z22, 1z2a) [18]. Often non-glycine substitutions at this position are conserved across an entire subfamily whose members span distinct phyla. For example, an alanine substitution is conserved across the Rab32 subfamily [19] whose members span at least eight phyla. Thus such (relatively rare) substitutions appear to perform a functional role specific to these subfamilies.

## Discussion

Because the glycine brace is the single structural feature that most distinguishes Rab-related GTPases from other Ras-like GTPases (Figure 2B), it presumably plays a critical functional role somehow related to the conserved atomic interactions described above. Given that the guanine-binding loop and the P-loop bind to both ends of GTP or GDP, the glycine brace could promote guanine nucleotide binding by stabilizing the conformations of these glycines, which could serve as hinges for opening and closing of these loops. Conversely, disruption of these aromatic-glycine interactions could promote the release of GDP during nucleotide exchange. It is worthwhile noting in this context that the most buried residue (164 Ǻ2) of Ran GTPase upon binding to its nucleotide exchange factor, RCC1 [20], is a lysine that is located near the center of the glycine brace α helix (Lys99-Ran in Figure 3D). Moreover, in the Ran-RCC1 crystal structure this lysine is inserted into the central hole of RCC1's β-propeller domain whereas the CH-π and NH-π interactions between the conserved tryptophan and the P-loop glycine are disrupted (compare Trp104-Ran in Figures 3C and 3D); taken together, this suggests a possible role for the glycine brace in nucleotide exchange within Ran GTPases.

What role might the non-glycine substitutions preceding the guanine-binding loop perform? To address this question, it should be noted that alanine is much more likely to occur as a substitute for glycine at this position than are other residues; this can be explained by the fact that both glycine and alanine promote structural flexibility [21-23]. However, as indicated by their Ramachandran plots, alanine is less flexible than glycine, suggesting that an alanine substitution decreases somewhat the flexibility of the guanine-binding loop. Perhaps turning on these alanine-variant GTPase switches at inappropriate times is highly detrimental, and, as a result, nucleotide exchange is suppressed (relative to other GTPase switches) by having a less flexible guanine-binding loop. Similarly, a non-glycine, non-alanine substitution seems likely to decrease the flexibility of the guanine-binding loop more dramatically; in these cases, the participation of specific exchange factor interactions may be required for nucleotide release leading to even more stringent, pathway-specific regulation.

Co-conservation of the glycine brace with the threonine and alanine of the Walker B (DTAG) motif (Figure 2) also is consistent with a role for the glycine brace in nucleotide exchange. Repositioning of the alanine is proposed to facilitate nucleotide exchange by occluding the Mg++ binding site, leading to expulsion of the phosphate-associated Mg++ ion [13]. Co-conservation of the glycine brace with this alanine thus suggests the possibility that all six of the Rab-related residues highlighted in Figure 2B somehow function as a unit to regulate nucleotide binding and release.

## Conclusion

It is proposed that the two glycine residues, one preceding the guanine-binding loop and another preceding the P-loop, function as hinges and that the glycine brace influences guanine nucleotide binding or release by interacting with these hinges. This has obvious implications regarding the regulation of Rab-related GTPase switches via guanine nucleotide exchange. Of course, the precise manner in which the glycine brace might play a role in nucleotide exchange remains to be determined.

## Methods

P loop GTPases sequences were identified within the NCBI nr, env_nr and translated EST databases using PSI-BLAST [24] and motif-based [25] search procedures. These sequences were multiply aligned using a variety of methods, including: manual curation of PSI-BLAST checkpoint files in conjunction with the PSI-BLAST alignment algorithm, MUSCLE [26,27], Bayesian sequence alignment methods [25,28,29], and the CE structurally-based alignment method [30]. Manual curation was performed in conjunction with structural analysis of sequence patterns using the CHAIN program [12]. Aligned sequences were partitioned into functionally divergent subgroups using a Bayesian partitioning with pattern selection (BPPS) procedure [11]; this identified both the Ras-like (Figure 1B) and Rab-related (Figure 2B) subgroups. The BPPS procedure is implemented within the CHAIN program [12]; for a review of CHAIN analysis see [31]. The Reduce program [32] was used to add hydrogen atoms to structural coordinate files. Molecular images were created by applying the Rasmol program [33] to the following structural coordinate files (pdb identifiers): 1g17[34], 1oiw[35], 1i4t[36], 1byu[37], and 1i2m[20].
